# Supplementary material for: Role of air-sea heat flux on the transformation of Atlantic Water encircling the Nordic Seas
Source: Nat Commun. 2023 Jan 10;14:141. doi: 10.1038/s41467-023-35889-3 (PMC9832023; doi:10.1038/s41467-023-35889-3)
Supplement: Supplementary file 1 — Supplementary Information [file 41467_2023_35889_MOESM1_ESM.pdf]

Supplementary Information

Role of air-sea heat flux on the transformation of Atlantic Water encircling the Nordic Seas

Jie Huang[1*], Robert S. Pickart[1], Zhuomin Chen[2], Rui Xin Huang[1]

[1] Woods Hole Oceanographic Institution, Woods Hole, MA, USA

[2] Department of Marine Sciences, University of Connecticut, Groton, CT, USA

*Corresponding author: jhuang@whoi.edu

Contents of this file

Supplementary Tables 1-2

Supplementary Figures 1-6

Supplementary Table 1 | Data sources with corresponding time periods and references.

Data description	Data source and reference	Years of data used in this study	Years of original dataset
A comprehensive historical hydrographic dataset	Access to the individual historical hydrographic data source is listed in the Supplementary Table 1 of Huang et al. (2020). The combined hydrographic dataset is available on request from Dr. Ailin Brakstad (Ailin.Brakstad@uib.no)	1993-2018	1980-2018
3-D velocity field from GLORYS12 reanalysis product	Data website page: https://resources.marine.copernicus.eu/product-detail/GLOBAL_MULTIYEAR_PHY_001_030/INFORMATION.	1993-2018	1993-2019
Sea surface geotrophic velocity from satellite observations (CMEMS)	Data website page: https://resources.marine.copernicus.eu/product-detail/SEALEVEL_GLO_PHY_L4_MY_008_047/INFORMATION.	1993-2018	1993-present
ADCP velocity from historical cruises	1) JCR 2012 curise, Håvik et al. (2012),<br>data website page: http://kogur.whoi.edu/php/index.php.<br>2) PROVOLO 2016-2017 cruise, Bosse et al. (2019),<br>data website page: http://metadata.nmdc.no/metadata-api/landingpage/70e5d6d3a27165e7f82bc764c5676937.<br>3) IGP 2018 cruise, Huang et al. (2021),<br>data website page: https://web.whoi.edu/all0118/.	2012, 2016, 2017 and 2018	2012, 2016, 2017 and 2018
ADCP velocity from historical moorings	1) Svinøy transect moorings 2005-2015, provided by Dr. Kjell Arild Orvik, data website page: http://metadata.nmdc.no/UserInterface/#/.<br>2) PROVOLO 2016-2017 moorings, Fer et al. 2020,<br>data website page: http://metadata.nmdc.no/metadata-api/landingpage/18be4af4b59b871127a174bde88d50a1.	2005-2017	2005-2017
ADCP velocity from historical gliders	Lofoten basin 2012-2014 and PROVOLO 2016-2017 gliders, from Norgliders, GFI, University of Bergen,<br>data website page: https://norgliders.gfi.uib.no/page/figs.html.	2012-2014 and 2016-2017	2012-2014 and 2016-2017
ERA5 air-sea heat flux and wind stress data	Data website page: https://www.ecmwf.int/en/forecasts/datasets/reanalysis-datasets/era5.	1993-2018	1979-present
3-D velocity field from ARMOR3D reanalysis product	Data website page: https://data.marine.copernicus.eu/product/MULTIOBS_GLO_PHY_TSUV_3D_MYNRT_015_012/description	2005-2018	1993-present
3-D velocity field from ASTE reanalysis product	Data website page: https://web.corral.tacc.utexas.edu/OceanProjects/ASTE/	2005-2017	2002-2017
3-D velocity field from GREP reanalysis product	Data website page: https://data.marine.copernicus.eu/product/GLOBAL_REANALYSIS_PHY_001_026/description).	2005-2018	1993-present

Supplementary Table 2 | Net cooling of Atlantic Water along the two Norwegian Atlantic Current (NwAC) branches from the long-term mean of observations, from the ensemble mean of monthly Price-Weller-Pinkel (PWP) simulations, and their difference. A linear fit is used to obtain the net cooling along the entire current. The error in observations indicates the standard error from the linear fit. The error in PWP model was estimated by considering an uncertainty of 0.02 m s^{-1} for the alongstream velocity of the current.

	Observations (net cooling)	PWP model (net air-sea cooling)	Difference between observations and model (net lateral cooling)
NwAFC	-2.2±0.2 °C	-2.4±0.6 °C	+0.2±0.6 °C
NwASC	-3.3±0.2 °C	-0.7±0.6 °C	-2.6±0.6 °C

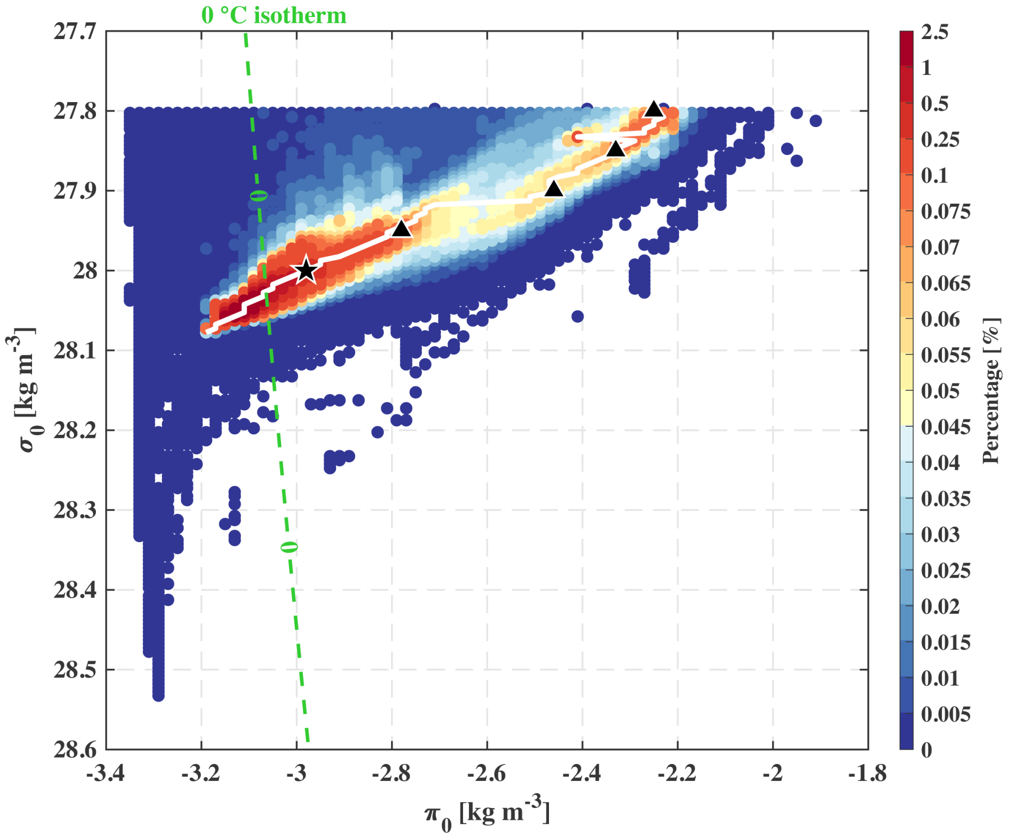

Supplementary Figure 1. Percent occurrence of dense water in σ_0-π_0 space (where σ_0 and π_0 are the potential density and potential spicity, respectively, referenced to the sea surface), using the historical hydrographic data (2005-2018) from the upper 850m of Nordic Seas. The white line indicates the largest occurrence percentages for the density range 27.8 to 28.05 kg m^{-3}, with an interval of 0.005 kg m^{-3}. The dense Atlantic Water mode used to compute the σ_0-π_0 distance in Figure 1c is indicated by the black star (σ_0 = 28.0 kg m^{-3}, π_0= -2.98 kg m^{-3}). The black triangles are less-dense modes that were considered (see text). The green dashed line indicates the 0 °C isotherm, which is the commonly accepted division between Atlantic-origin and Arctic-origin dense waters.

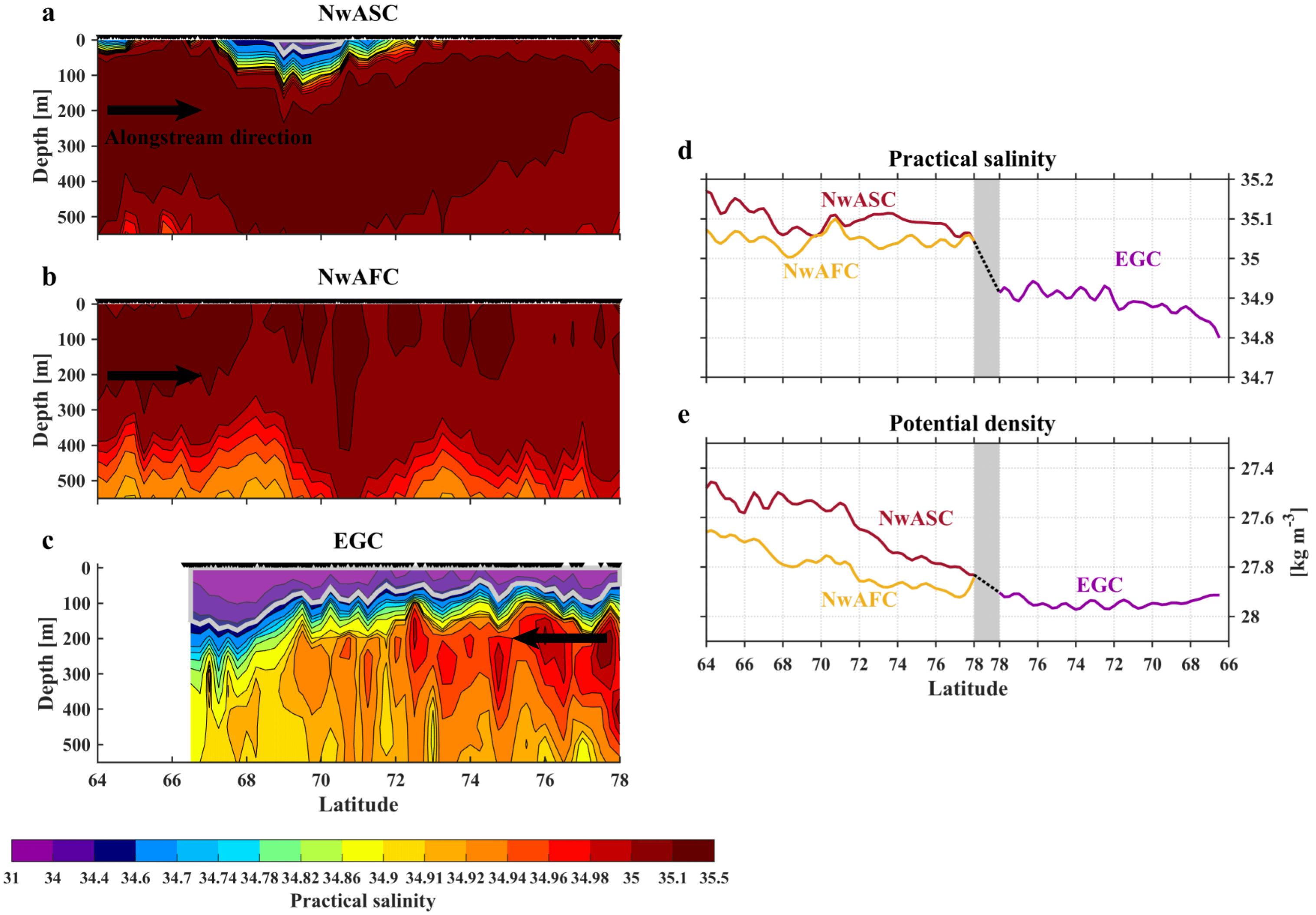

Supplementary Figure 2. Same as Figure 2 except for salinity. The Polar Surface Water layer (defined by salinity < 34.5 and potential density < 27.7 kg m^{-3}) is delimited by the thick gray contour.

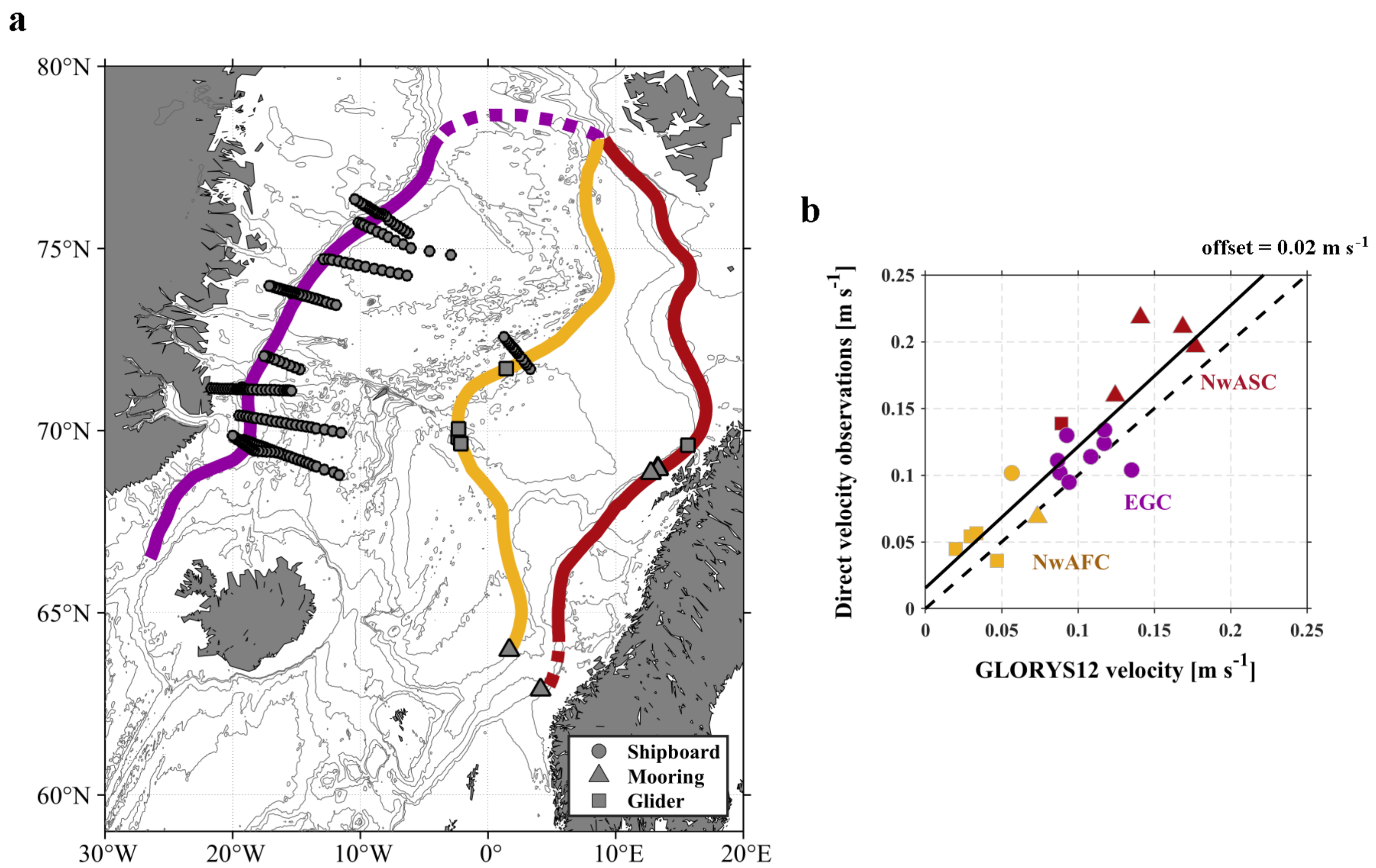

Supplementary Figure 3. Comparison between the long-term mean GLORYS12 reanalyzed velocity (2005-2018) and historical velocity observations. (a) The spatial distribution of shipboard, mooring, and glider data (see the legend). (b) Comparison between observed and reanalyzed alongstream velocity, with the lines of linear fit (solid line, P value in *t-test* < 0.05) and perfect fit (dashed line) indicated. The Norwegian Atlantic Slope Current (NwASC), Norwegian Atlantic Front Current (NwAFC), and East Greenland Current (EGC) observations are shown in red, yellow and purple, respectively. The shipboard, mooring, and glider data are indicated by circles, triangles, and squares. The comparison was made by calculating the depth-mean velocity of the observations and reanalysis over the same depth ranges (0-550 m for the shipboard measurements, 100-500 m for the mooring measurements, and 0-1000 m for the glider measurements). The data sources and time periods of the historical velocity observations are listed in Supplementary Table 1. The bathymetry (contours) is the same as in Figure 1.

a

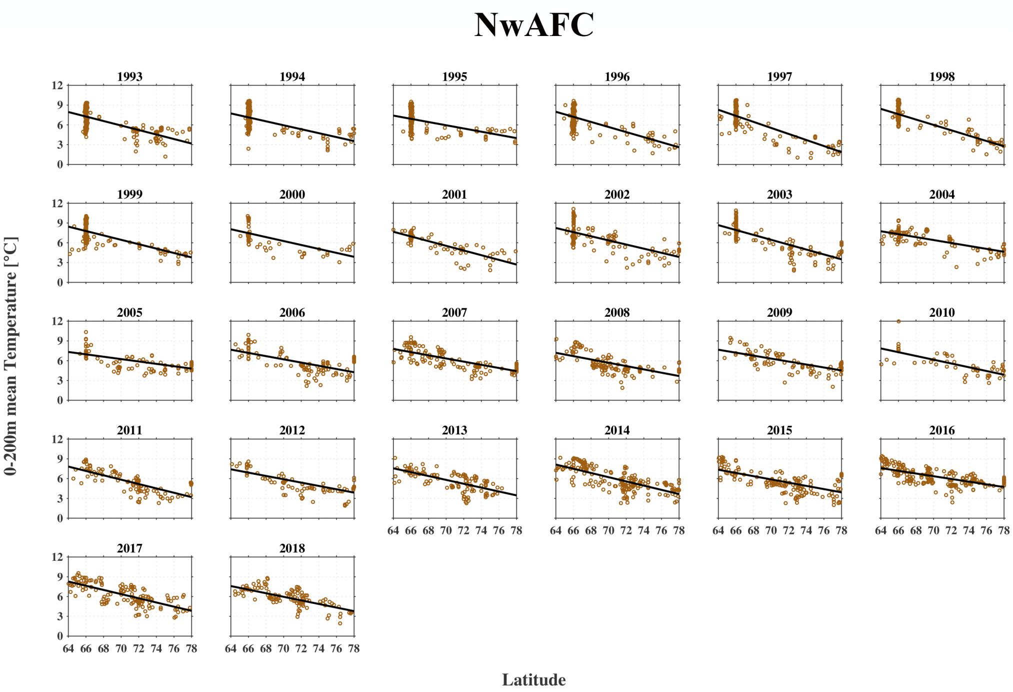

b

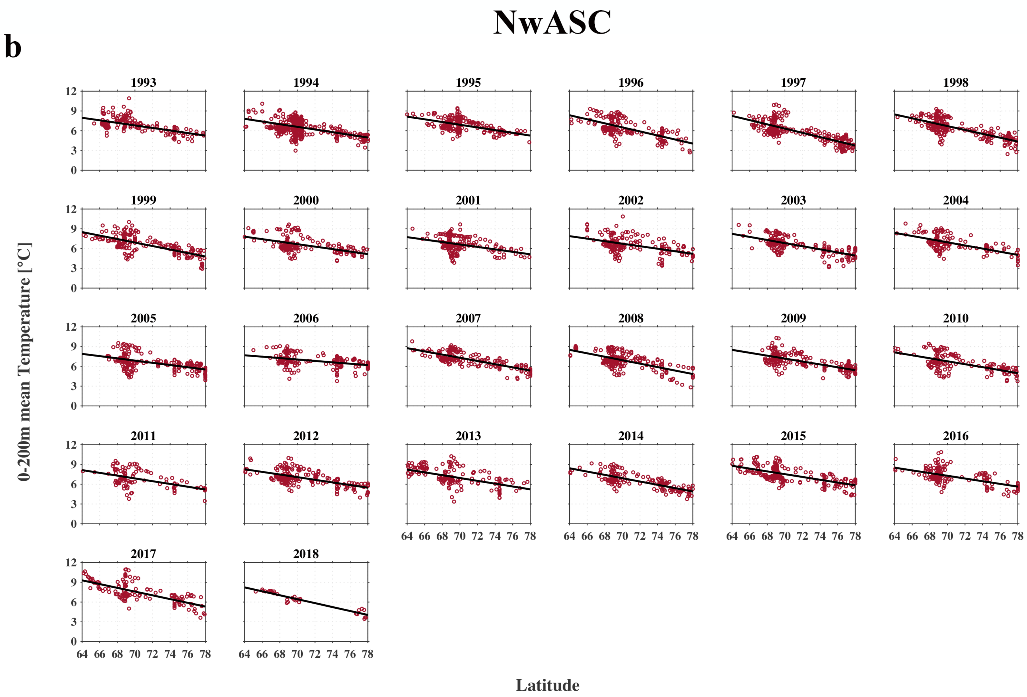

Supplementary Figure 4. The linear fits ($y = a \cdot x + b$, where x is Latitude and y is 0-200m mean temperature) used to obtain the net cooling of Atlantic Water based on the historical data (circles; see text for details). The net temperature change is taken to be $a \cdot \Delta x$, where a is the slope of the linear fit, and $\Delta x = 78°N - 64°N$. (a) Norwegian Atlantic Front Current (NwAFC), and (b) Norwegian Atlantic Slope Current (NwASC), where each panel is for the given year from 1993 to 2018.

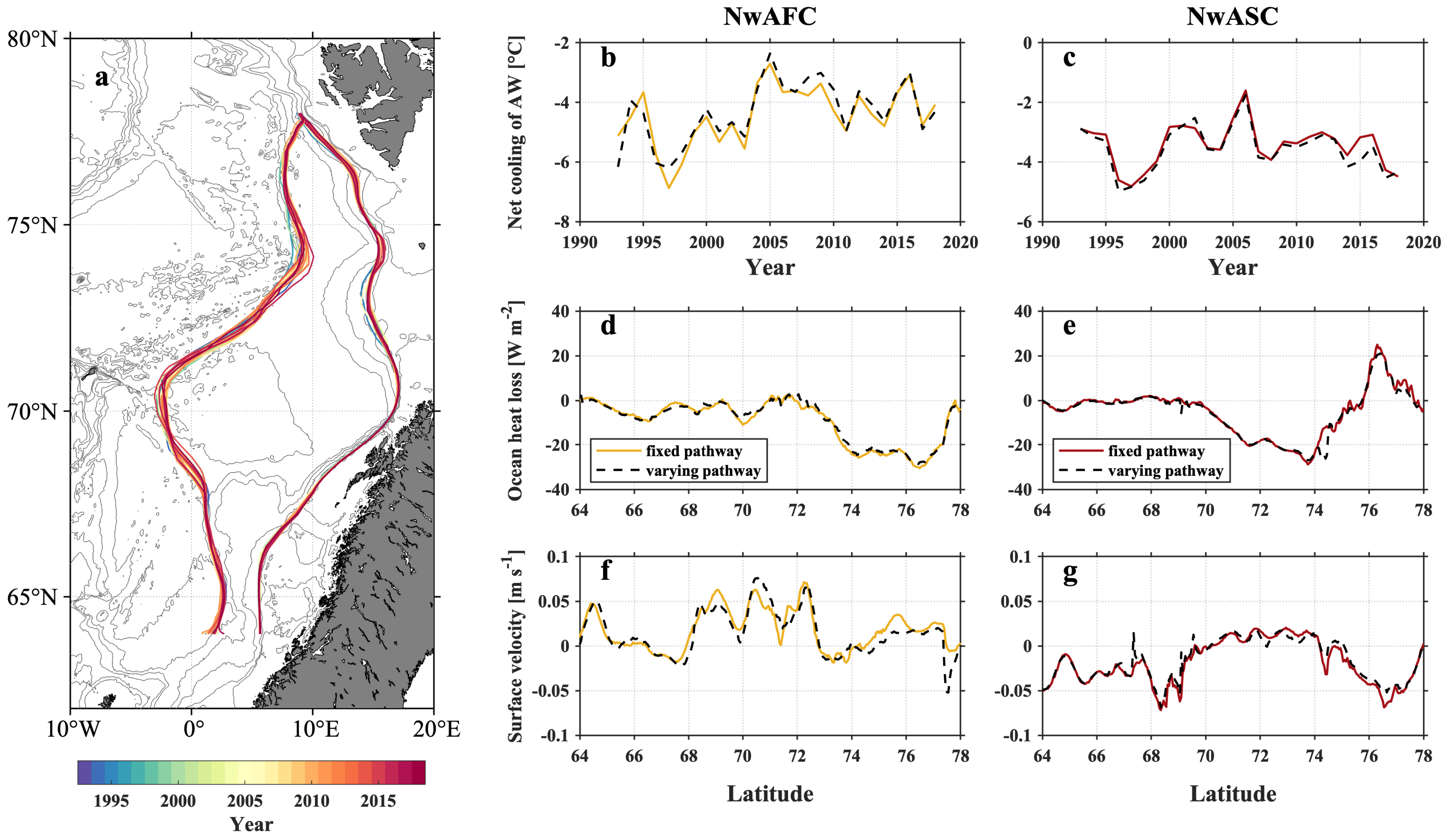

Supplementary Figure 5. (a) Yearly pathways (defined by the maxima of velocity in each year) of two Norwegian Atlantic Current (NwAC) branches from 1993 to 2018. The remaining panels show the comparison of the long-term changes of (b and c) net cooling of Atlantic Water (AW); (d and e) air-sea turbulent heat flux; and (f and g) surface velocity obtained by using long-term mean fixed pathways (solid lines) and yearly varying pathways (dashed lines). We note that when considering the yearly varying pathways (e.g., the NwAFC), we firstly computed the along-pathway variations of temperature, turbulent heat loss and surface velocity for each year (as a function of latitude), using the data (within the 50 km swath) and pathway in the corresponding year. Then, a linear fit was applied at each latitude to obtain the long-term changes along the pathway. The bathymetry (contours) is the same as in Figure 1.

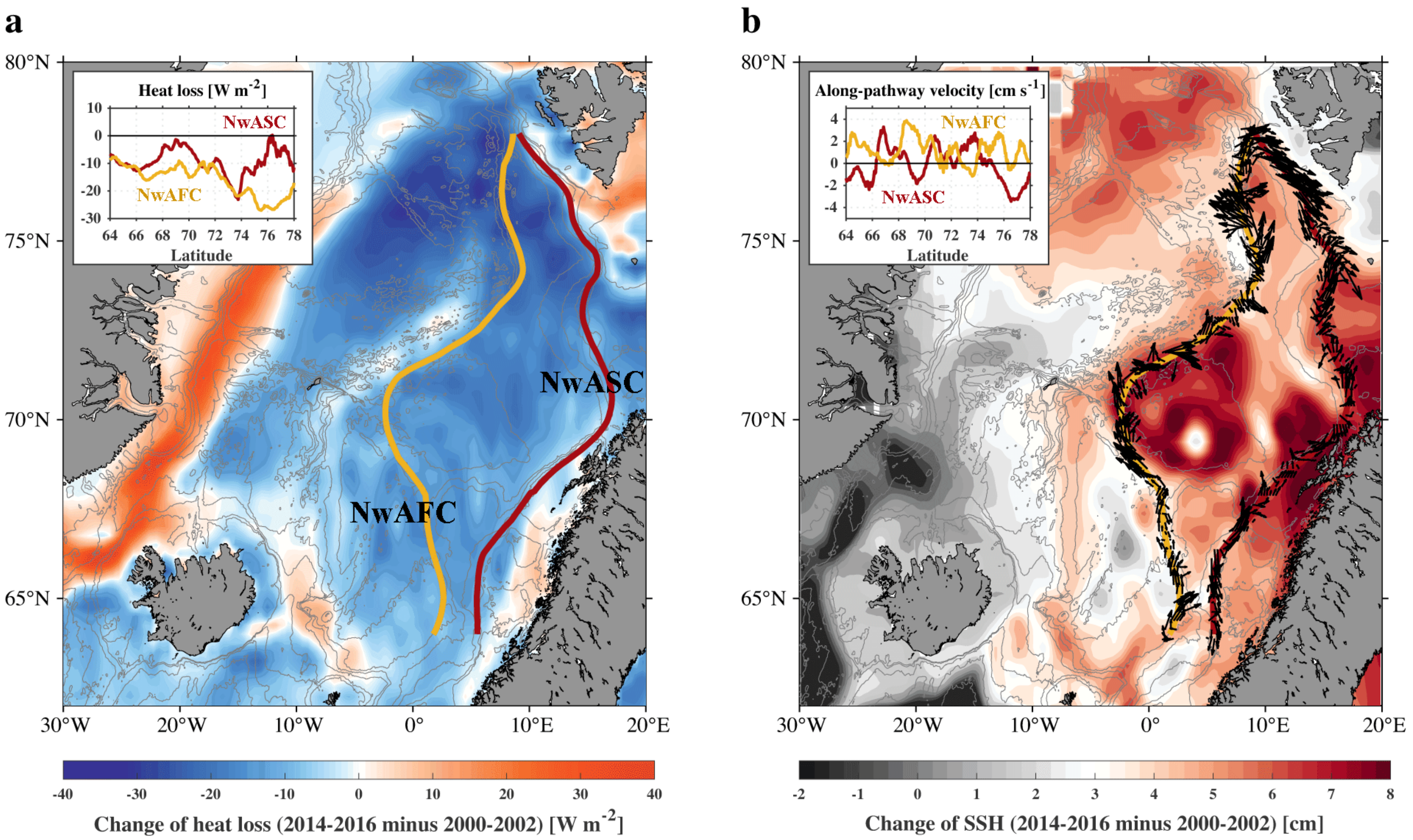

Supplementary Figure 6. Change of year-round mean (a) air-sea turbulent heat loss; and (b) sea surface height (color) and sea surface velocity (vectors) between early period 2000-2002 and late period 2014-2016 (the difference is obtained using the 2014-2016 mean minus the 2000-2002 mean). The changes along the pathways of the Norwegian Atlantic Front Current (NwAFC, yellow) and Norwegian Atlantic Slope Current (NwASC, red) are shown in the subfigures in the upper left. The sea surface velocity and sea surface height (SSH) data are from GLORYS12 and the satellite observations, respectively. The bathymetry (contours) is the same as in Figure 1.
